# Postoperatives Management nach dekompressiver Hemikraniektomie bei malignem Mediainfarkt – eine deutschlandweite Umfragestudie

**DOI:** 10.1007/s00115-023-01486-4

**Published:** 2023-05-04

**Authors:** D. Schoene, C. Hartmann, S. Winzer, H. Moustafa, A. Günther, V. Puetz, K. Barlinn, IGNITE study group

**Affiliations:** 1 Klinik und Poliklinik für Neurologie, Medizinische Fakultät und Universitätsklinikum Carl Gustav Carus, Technische Universität Dresden, Fetscherstr. 74, 01307 Dresden, Deutschland; 2 Dresdner Neurovaskuläres Centrum, Medizinische Fakultät und Universitätsklinikum Carl Gustav Carus, Technische Universität Dresden, Dresden, Deutschland; 3 Institut und Poliklinik für Diagnostische und Interventionelle Neuroradiologie, Medizinische Fakultät und Universitätsklinikum Carl Gustav Carus, Technische Universität Dresden, Dresden, Deutschland; 4 Klinik für Neurologie, Universitätsklinikum Jena, Jena, Deutschland

**Keywords:** Raumfordernder Hirninfarkt, Neurointensivtherapie, Entlastungstrepanation, Therapeutische Sedierung, Umfragestudie, Large hemispheric infarction, Neurocritical care, Decompressive trepanation, Therapeutic sedation, Survey study

## Abstract

**Hintergrund:**

Der maligne Mediainfarkt ist ein potenziell lebensbedrohliches Krankheitsbild. Die dekompressive Hemikraniektomie gehört zur leitliniengerechten Behandlungspraxis insbesondere bei Patienten/-innen bis zu 60 Jahren. Für das postoperative Management gibt es keine standardisierte Handlungsempfehlung.

**Ziel der Arbeit (Fragestellung):**

Die Untersuchung zielt darauf ab, die gegenwärtige Versorgungslage hinsichtlich der Anwendung standardisierter Behandlungskonzepte zu analysieren und Impulse für eine optimierte Versorgung von Patienten/-innen mit einem malignen Mediainfarkt im neurointensivmedizinischen Bereich zu generieren.

**Material und Methoden:**

Vom 20.09.2021 bis zum 31.10.2021 wurden 43 Mitglieder des Netzwerks Initiative of German NeuroIntensive Trial Engagement (IGNITE) eingeladen, an einer standardisierten anonymen Onlineumfrage teilzunehmen. Es erfolgte eine deskriptive Datenanalyse.

**Ergebnisse:**

Neunundzwanzig von 43 Zentren (67,4 %) nahmen an der Umfrage teil, davon 24 Universitätskliniken. Über eine eigenständige neurologische Intensivstation verfügen 21 Krankenhäuser. Während 23,1 % ein standardisiertes Vorgehen bei der postoperativen Analgesie und Sedierung favorisieren, werden in der Mehrzahl individuell gewählte Kriterien hinzugezogen (Einschätzung der Zunahme des intrakraniellen Druckes, Weaningparameter, Komplikationen). Der Zeitpunkt der angestrebten Extubation variiert zwischen den Kliniken (≤ 24 h bei 19,2 %, ≤ 3 Tage bei 30,8 %, ≤ 5 Tage bei 19,2 %, > 5 Tage bei 15,4 %). Eine Frühtracheotomie (≤ 7 Tage) wird bei 19,2 % der Kliniken durchgeführt. Intravenöse Osmotherapeutika werden bei 53,9 % standardisiert angewandt. Zweiundzwanzig Zentren (84,6 %) erklärten sich bereit, an einer klinischen Studie zur Dauer der postoperativen Sedierung und Beatmung teilzunehmen.

**Diskussion:**

Die Ergebnisse zeigen eine bemerkenswerte Heterogenität in der Behandlungspraxis von Patienten/-innen mit malignem Mediainfarkt und insbesondere der Dauer der postoperativen Analgesie und Sedierung sowie Beatmung nach erfolgter Hemikraniektomie in Deutschland. Die Durchführung einer randomisierten Studie zur Sedierungsdauer nach Hemikraniektomie scheint gerechtfertigt.

**Zusatzmaterial online:**

Die Onlineversion dieses Beitrags (10.1007/s00115-023-01486-4) enthält zusätzliches Material (Umfrage). Beitrag und Zusatzmaterial stehen Ihnen auf www.springermedizin.de zur Verfügung.

## Abstract (en)

**Background:**

Malignant middle cerebral artery infarction is a potentially life-threatening disease. Decompressive hemicraniectomy constitutes an evidence-based treatment practice, especially in patients under 60 years of age; however, recommendations with respect to postoperative management and particularly duration of postoperative sedation lack standardization.

**Objective:**

This survey study aimed to analyze the current situation of patients with malignant middle cerebral artery infarction following hemicraniectomy in the neurointensive care setting.

**Material and methods:**

From 20 September 2021 to 31 October 2021, 43 members of the initiative of German neurointensive trial engagement (IGNITE) network were invited to participate in a standardized anonymous online survey. Descriptive data analysis was performed.

**Results:**

Out of 43 centers 29 (67.4%) participated in the survey, including 24 university hospitals. Of the hospitals 21 have their own neurological intensive care unit. While 23.1% favored a standardized approach regarding postoperative sedation, the majority utilized individual criteria (e.g., intracranial pressure increase, weaning parameters, complications) to assess the need and duration. The timing of targeted extubation varied widely between hospitals (≤ 24 h 19.2%, ≤ 3 days in 30.8%, ≤ 5 days in 19.2%, > 5 days in 15.4%). Early tracheotomy (≤ 7 days) is performed in 19.2% and 80.8% of the centers aim for tracheotomy within 14 days. Hyperosmolar treatment is used on a regular basis in 53.9% and 22 centers (84.6%) agreed to participate in a clinical trial addressing the duration of postoperative sedation and ventilation.

**Conclusion:**

The results of this nationwide survey among neurointensive care units in Germany reflect a remarkable heterogeneity in the treatment practices of patients with malignant middle cerebral artery infarction undergoing hemicraniectomy, especially with respect to the duration of postoperative sedation and ventilation. A randomized trial in this matter seems warranted.

Der maligne Mediainfarkt ist ein potenziell lebensbedrohliches Krankheitsbild. Neben der Akutversorgung und Diagnosestellung ist die frühe Einschätzung der Schwellungsdynamik von hoher Relevanz, um eine individuelle Entscheidung zur operativen Hemikraniektomie stellen zu können. Für das postoperative Management und insbesondere die Dauer der postoperativen Analgesie und Sedierung sowie Beatmung nach Hemikraniektomie sind keine evidenzbasierten Daten verfügbar. In der vorliegenden Umfrage deutscher Neurointensivstationen wird die aktuelle Praxis vorgestellt und diskutiert.

## Hintergrund und Ziel

**Figure 1:**
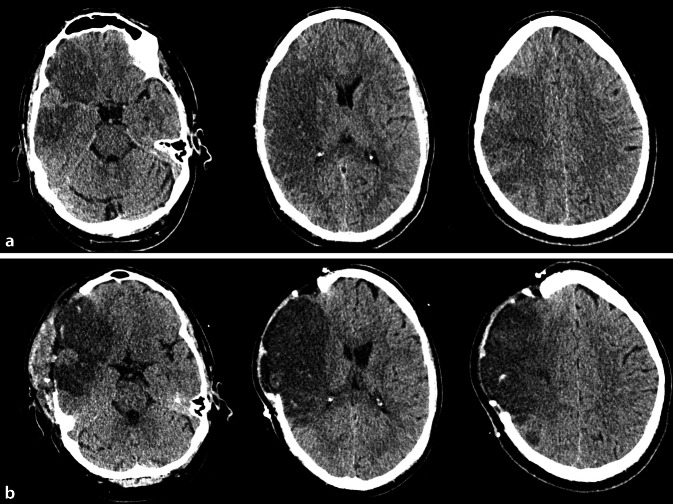

Der maligne Mediainfarkt tritt bei bis 10 % der Patienten/-innen mit ischämischem Schlaganfall auf und ist mit einer Letalität von 70 % unter konservativer Therapie ein potenziell lebensbedrohliches Krankheitsbild [18]. Als Maximalvariante einer großflächigen Ischämie im A.-cerebri-media-Versorgungsgebiet ist der maligne Mediainfarkt Folge eines Verschlusses der intrakraniellen A. carotis interna oder der proximalen A. cerebri media, fakultativ mit Beteiligung der benachbarten Gefäßterritorien (A. cerebri anterior, A. cerebri posterior). Klinisch besteht ein schwerwiegendes Mediasyndrom. Neben einem National-Institutes-of-Health-Stroke-Scale(NIHSS)-Score von ≥ 15 Punkten ist die Vigilanzminderung ein klinisches Kernkriterium. Da die Gesamtausdehnung des Ödems von der Infarktgröße abhängt, kommt es beim malignen Mediainfarkt zu einem progredient raumfordernden Effekt mit Verschiebung der intrakraniellen Kompartimente und Zunahme des intrakraniellen Druckes mit drohender sekundärer Infarzierung vitalen Hirngewebes und lebensbedrohlicher Herniation (Abb. 1; [23]). Dabei ist zu betonen, dass die Vigilanzminderung als klinisches Indiz für eine Erhöhung des intrakraniellen Druckes meist die Folge bereits dekompensierter intrakranieller Reservevolumina ist. Die frühe Einschätzung der individuell unterschiedlichen Schwellungsdynamik ist zwingend notwendig, um eine frühe therapeutische Entscheidung bezüglich einer dekompressiven Hemikraniektomie treffen zu können [8].

Die dekompressive Hemikraniektomie gehört mittlerweile zu einer leitliniengerechten Behandlungspraxis bei Patienten/-innen im Alter ≤ 60 Jahren und steht als oftmals einzige lebensrettende Maßnahme zur Verfügung [13, 23, 25]. Die gepoolte Analyse dreier europäischer randomisierter kontrollierter Studien (DECIMAL, DESTINY, HAMLET) hat gezeigt, dass die Hemikraniektomie bei Patienten/-innen bis zu 60 Jahren sowohl zu einer Verbesserung der Überlebenschancen als auch zur Verbesserung des neurologischen Funktionsniveaus führt. Eine randomisierte kontrollierte Folgestudie (DESTINY-II) konnte zudem zeigen, dass auch Patienten/-innen über 60 Jahre bezüglich des funktionellen Outcomes entsprechend einer modifizierten Rankin-Scale (mRS) von 0–4 (38 % vs. 18 %, *p* = 0,04) von einer Hemikraniektomie profitieren, allerdings bei der Mehrheit mit schweren funktionellen Einschränkungen (mRS von 4 oder 5 Punkten; d. h. nicht selbständig gehfähig bzw. bettlägerig; [7, 18]). Neben der medizinischen Indikation müssen der (mutmaßliche) Patientenwille, das Alter, der prämorbide Zustand, Vorerkrankungen und das Rehabilitationspotenzial in die Entscheidung einbezogen werden [26]. Im Fokus steht dabei die Frage des Überlebens und des erwartbaren vs. akzeptablen funktionellen Outcomes [24].

Im Jahr 2015 wurden in einer Konsenskonferenz der Neurocritical Care Society (NCS) und der Deutschen Gesellschaft für NeuroIntensiv- und Notfallmedizin (DGNI) Empfehlungen zu wichtigen Elementen einer intensivmedizinischen Therapie des malignen Mediainfarkts erarbeitet. Diese beziehen sich neben Sedierung, Analgesie und Atemwegsmanagement auch auf Dysphagiediagnostik und Behandlung von Magen-Darm-Funktionsstörungen, Kontrolle des Blutzuckerspiegels und des Hämoglobinwertes, Thromboseprophylaxe und Sekundärprophylaxe, Barbiturat- und Osmotherapie, Temperatur- und Blutdruckmanagement und Optimierung der Lagerung [33]. Die spezifische postoperative Intensivtherapie, wie die Fortführung einer therapeutischen Sedierung mit dem Ziel der Kupierung eines erhöhten intrakraniellen Druckes und deren Dauer, ist darüber hinaus weiterhin unzureichend definiert. In einer deutschlandweit heterogenen Versorgungslandschaft besteht diesbezüglich weiterhin eine wissenschaftliche Evidenzlücke. Die aktuelle Umfrage deutscher Neurointensivstationen zielte darauf ab, die gegenwärtige Lage zu analysieren, um Impulse für eine standardisierte optimierte Versorgung von Patienten/-innen mit einem raumfordernden Mediainfarkt im neurointensivmedizinischen Bereich generieren zu können. Diese umfasst die postoperative Behandlungsstrategie zur Verbesserung des funktionellen Outcomes und Vermeidung potenzieller Komplikationen, welche mit einer Übersedierung, prolongierten Aufwachreaktion und prolongiertem Weaning oder auch unzureichender Therapie des intrakraniellen Druckes einhergehen können.

## Methoden

**Figure 2:**
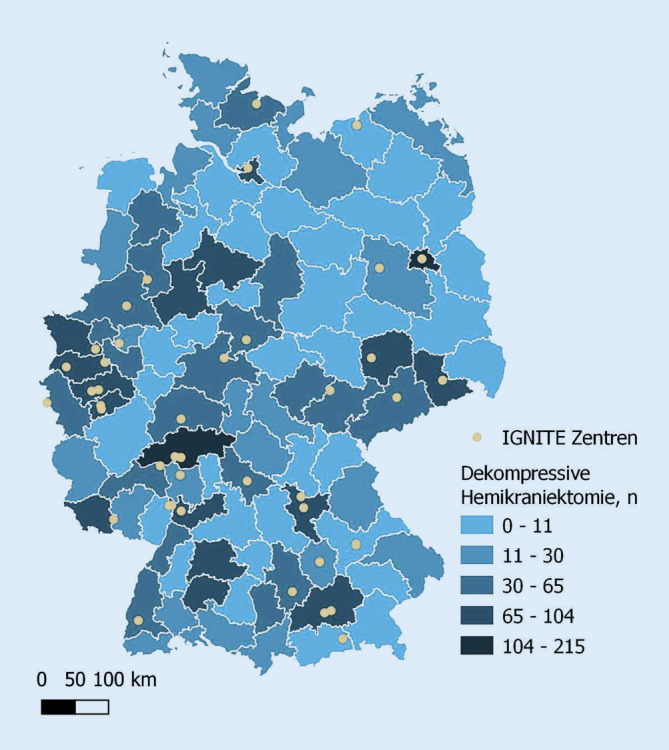

Im September 2021 wurden die Mitglieder des Netzwerks Initiative of German NeuroIntensive Trial Engagement (IGNITE) per E‑Mail eingeladen, an einer standardisierten anonymen Onlineumfrage teilzunehmen (Abb. 2). Das IGNITE-Netzwerk als Forschungsinitiative der DGNI umfasst eine Gruppe klinisch-wissenschaftlich aktiv tätiger Neurointensivmediziner/-innen (darunter Oberärzte/-innen und Leiter/-innen von Neurointensivzentren) in Deutschland mit dem Ziel, gemeinsame Forschungsprojekte im Bereich der Neurointensivmedizin zu initiieren [14]. Der Fragebogen beinhaltet insgesamt 39 Fragen (geschlossene Fragen mit Einfachnennung, halboffene und offene Fragen) zu den Themenbereichen Organisationsstruktur der Klinik (6), Versorgungssituation (6) und Indikationsstellung (5) einer dekompressiven Hemikraniektomie sowie Fragen zum chirurgischen Vorgehen (4) und zur postoperativen Intensivtherapie nach Hemikraniektomie mit Fokus auf Sedierung, Intubationsdauer, Verwendung einer ICP(intrakranieller Druck)-Sonde, Kontrollbildgebung, Osmotherapie und therapeutische Kühlung (17). Auch wurde nach der Bereitschaft zur Teilnahme an einer randomisierten Studie zur postoperativen Dauer einer therapeutischen Analgesie und Sedierung (1) gefragt. Der vollständige Fragebogen ist im Onlinezusatzmaterial abrufbar.

Die einzelnen Fragestellungen basierten als Bestandsaufnahme auf prozeduralen Kernpunkten und wurden auf Grundlage hausinterner Expertenstandards erstellt. Die Umfrage wurde über einen Zeitraum von 6 Wochen (20.09.2021 bis 31.10.2021) durchgeführt. Diese Fristsetzung wurde gewählt, um eine hinreichende Zeitspanne für die Beantwortung der Fragen bei parallel geringem Risiko für eine Verzerrung der Ergebnisse durch zeitabhängige Störvariablen zu ermöglichen. Die Auswertung erfolgte nach deskriptiver Analyse der Datensätze. Diese wurden nach webbasierter Erfassung (LimeSurvey, LimeSurvey GmbH, Hamburg, Deutschland) anonymisiert in einer Tabelle (Microsoft Excel, Version 16.65, Redmond, WA, USA) erfasst. Zur Analyse wurde IBM SPSS Statistics (Version 28.0.1.0, IBM Corp., Armonk, NY, USA) verwendet. Für kontinuierliche Variablen wurden der Median und der Interquartilsabstand (IQA) berechnet. Kategoriale Variablen wurden in relativen Häufigkeiten und Prozentangaben ausgewertet. Fehlende Werte in den jeweiligen Themenbereichen der Umfrage wurden in der Analyse nicht berücksichtigt.

Um das Ergebnis der Umfrage in Relation zur der Versorgungsstruktur in Deutschland zu stellen, wurden anhand der Qualitätszahlen des gemeinsamen Bundesausschusses (G-BA) unabhängig vom IGNITE-Netzwerk die Krankenhäuser in Deutschland identifiziert, welche mindestens 250 Patienten/-innen mit einem ischämischen Schlaganfall (gemäß I63-Hauptdiagnose) im Jahr 2019 behandelten. Das Jahr 2019 wurde als Bezugsjahr herangezogen, um einer möglichen Verzerrung durch die COVID-19-Pandemie zu entgehen. Eine Analyse mit den Variablen bzw. Häufigkeiten dekompressiver Hemikraniektomien (gemäß OPS 5‑012.0) und ischämischer Schlaganfälle in den jeweiligen Kliniken erfolgte anhand einer bivariaten linearen Regression. Zudem wurden die Hemikraniektomien nach geografischer Verteilung mittels Verortung der identifizierten Kliniken innerhalb der Raumordnungsregionen der Bundesrepublik Deutschland analysiert. Die graphische Darstellung erfolgte mittels Choroplethenkarte. Die Auswertung und Darstellung der Qualitätsberichtsdaten erfolgte mittels Python 3.10.1. (Wilmington, DE, USA). Die Erstellung der Karte erfolgte mittels QGIS 3.22.1 (QGIS Development Team, 2022. QGIS Geographic Information System. Open Source Geospatial Foundation Project).

## Ergebnisse

### Organisationsstruktur und Versorgungssituation

**Figure 3:**
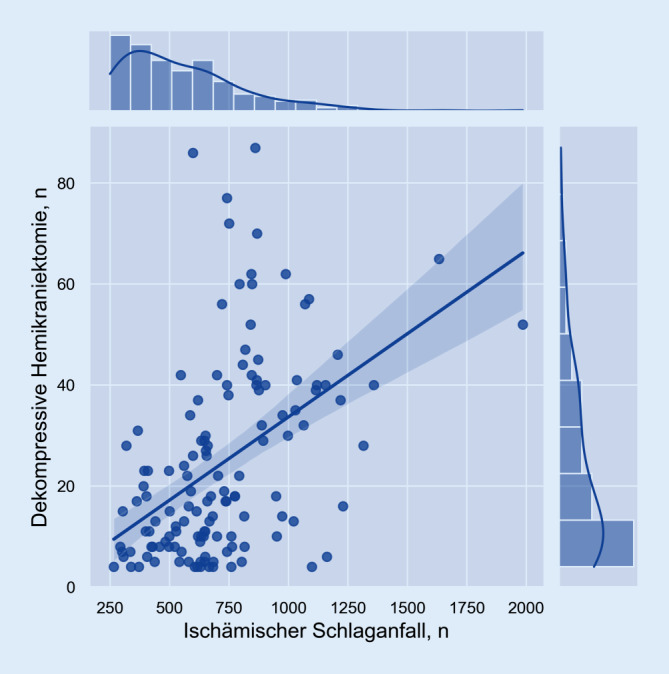

Anhand des Qualitätsberichtes des Gemeinsamen Bundesausschusses aus dem Jahr 2019 konnten gemäß Studienkriterien 456 Krankenhäuser identifiziert werden. Aufgrund datenschutzbedingter Verblindung der Eingriffszahlen wurden 245 (53,7 %) dieser Kliniken aus der Analyse exkludiert. In den verbleibenden 211 Kliniken wurden im Median 11 (IQA von 6–25) dekompressive Hemikraniektomien pro Jahr durchgeführt (Abb. 3). Die Regressionsanalyse weist auf einen Einfluss der Anzahl der versorgten ischämischen Schlaganfälle auf die Häufigkeit durchgeführter Hemikraniektomien pro Jahr hin (R^2^ = 0,218, *p* < 0,001; Abb. 3).

Die Zentren des IGNITE-Netzwerkes repräsentieren überwiegend Ballungsräume der Schlaganfallversorgung mit Durchführung von Hemikraniektomien (Abb. 3). Von den 43 angefragten Zentren des IGNITE-Netzwerks nahmen 29 Kliniken (67,4 %) an der Onlineumfrage teil. Davon entsprachen 24/29 (82,8 %) Krankenhäusern Universitätskliniken. Über eine eigenständige neurologische Intensivstation verfügten 21/29 (72,4 %) Krankenhäuser. Drei (10,3 %) der teilnehmenden Kliniken beantworteten nur einzelne Themenbereiche der Umfrage. Eine Übersicht zum Antwortverhalten befindet sich im Onlinezusatzmaterial (Tab. 1).

Die Bettenkapazität für die teilnehmenden neurologischen Kliniken liegt zwischen 40 und 120 Betten (Median 80, IQA 72–90). Für die Stroke-Units wurde eine Gesamtkapazität von 8 bis 26 Betten (Median 15, IQA 12–16) berichtet, wobei diese neben zertifizierten auch Enhanced-care-Betten beinhaltet. Die Bettenzahlen für die Intensivstationen, welche teilweise interdisziplinär belegt sind, schwanken zwischen 4 und 46 Betten (Median 12, IQA 9,8–12,3). Kliniken mit eigenständiger neurologischer Intensivstation stehen 8 bis 16 Betten (Median 12, IQA 10–12) zur Verfügung. Sämtliche an der Umfrage teilnehmende Kliniken haben eine angegliederte interventionelle Neuroradiologie vor Ort. Bis auf 2 Kliniken verfügen alle Kliniken über eine neurochirurgische Klinik.

Nach Selbsteinschätzung der beteiligten Kliniken besteht bei 60,7 % (17/28) Erfahrung mit der Betreuung von mehr als 12 Patienten/-innen mit malignem Mediainfarkt pro Jahr. Über 57,1 % (16/28) der Kliniken verfügen über eine hausinterne SOP.

### Indikationsstellung der Hemikraniektomie

Über 74,1 % (20/27) der Kliniken stellen die Indikation zur Hemikraniektomie anhand der DESTINY-Kriterien [7]. Für die Indikationsstellung einer Hemikraniektomie haben 74,1 % (20/27) keine obere Altersgrenze festgelegt. Bei 7 Kliniken, welche eine obere Altersgrenze anwenden, schwankt diese zwischen 60 und 80 Jahren (Median 70, IQA 60–75), wobei eine Klinik sich eine „individuelle Flexibilität“ vorbehält.

Über 77,8 % (21/27) der Kliniken halten an einem festgesetzten Zeitfenster ≤ 48 h nach Symptombeginn für die Durchführung einer Hemikraniektomie bei malignem Mediainfarkt fest, allerdings wird bei diesen Kliniken eine Hemikraniektomie in Ausnahmefällen auch außerhalb dieses Zeitfensters durchgeführt.

### Perioperatives Management – ICP-Sonde und Kontrollbildgebung

Nach Indikationsstellung zur Hemikraniektomie wird in 25,9 % (7/27) der Kliniken perioperativ eine ICP-Sonde angelegt. Eine Kontrollbildgebung wird standardisiert bei 44,4 % (12/27) unmittelbar postoperativ und bei 81,5 % (22/27) am Folgetag nach der Operation durchgeführt. Bei 33,3 % (9/27) der Kliniken erfolgt die Kontrollbildgebung nach Hemikraniektomie an beiden Tagen. Drei Kliniken gaben davon abweichende standardisierte Zeitpunkte für die Kontrollbildgebung innerhalb von 24 h bis zu Tag 5 an.

### Postoperatives Management – Analgesie, Sedierung und Beatmung

Für die Dauer der postoperativen Analgesie und Sedierung nach Hemikraniektomie gibt es bei 23,1 % (6/26) der Kliniken ein standardisiertes Vorgehen. Der standardisierte Zeitpunkt der Sedierungsrücknahme variiert dabei zwischen unmittelbarer Sedierungsrücknahme postoperativ nach Hemikraniektomie (*n* = 2), ≤ 24 h (*n* = 1), ≤ 3 Tagen (*n* = 2) und ≤ 5 Tagen (*n* = 1).

Für die Sedierungsrücknahme ohne standardisiertes Vorgehen, welche der überwiegende Teil der teilnehmenden Kliniken praktiziert, werden unterschiedliche Kriterien hinzugezogen – die Einschätzung der Zunahme des intrakraniellen Druckes (klinischer Verlauf einschließlich Reaktion auf Sedierungsrücknahme, gemessener ICP-Wert, Bildgebung, Neurosonographie), die Weaningparameter (Schutzreflexe, Tubustoleranz, Beatmungsprobleme) und erwartbare Komplikationen (Ausgangszustand, pulmonale Situation, Dysphagie, Auftreten akut symptomatischer Anfälle). Eine Kontrollbildgebung wird bei 46,2 % (12/26) regelhaft vor Rücknahme der Sedierung durchgeführt.

Zur Sedierung werden überwiegend (96,2 %, 25/26) intravenöse Sedativa verwendet, eine Klinik (3,9 %, 1/26) wendet bevorzugt inhalative Sedativa an. Es kommen hauptsächlich Propofol (88 %, 22/25), Midazolam (80,0 %, 20/25) und Ketamin 40 % (10/25) zum Einsatz, Thiopental wird nur im Einzelfall (4,0 %, 1/25) und Etomidat nicht angewandt. Zur Analgesie werden überwiegend Sufentanil (88 %, 22/25), aber auch Fentanyl (12 %, 3/25) und zu einem geringen Anteil Remifentanil (4 %, 1/25) verwendet. Adjuvant werden Alpha-2-Agonisten (Dexmedetomidin 40 % [10/25], Clonidin 24 % [6/25]) verabreicht.

### Osmotherapie und therapeutische Kühlung

Intravenöse Osmotherapeutika werden bei 53,9 % (14/26) der Kliniken nach Hemikraniektomie standardisiert zur medikamentösen Therapie zur Senkung des intrakraniellen Druckes angewandt. Davon wird bei 71,4 % (10/14) der Kliniken nach einem festen Schema vorgegangen. Die verwendeten Substanzen sind vorrangig Mannitol (78,6 %, 11/14) und hyperosmolares Natrium (64,3 %, 9/14). Eine Klinik (7,1 %, 1/14) verwendet ausschließlich Glycerosteril. Die Indikationsstellung erfolgt bei 5 Kliniken (41,7 %, 5/12) explizit auf Grundlage der Messung des intrakraniellen Druckes via ICP-Sonde.

In einem Zentrum (3,9 %, 1/26) wird eine therapeutische Kühlung nach Hemikraniektomie mit dem Ziel der Normothermie angewandt. Von den befragten Zentren hatten 23,1 % (6/26) an der Studie DEcompressive surgery Plus hypoTHermia for Space-Occupying Stroke (DEPTH-SOS) teilgenommen. In dieser randomisierten kontrollierten Studie wurde die Kombination von Hemikraniektomie und therapeutischer Hypothermie (33 ± 1 °C, über ≥ 72 h) untersucht, mit letztlich negativen Ergebnissen bezüglich der klinischen Endpunkte Mortalität und funktionelles Outcome [21].

### Weaning – Dauer bis Extubation oder Tracheotomie

Die Intubationsdauer, d. h. der Zeitraum, über den die Patienten/-innen postoperativ standardisiert intubiert bleiben, variiert. Bei 15,4 % (4/26) der Kliniken wird die Extubation in der Regel direkt nach erfolgter Entlastungsoperation geplant. Die Extubation wird bei 19,2 % (5/26) ≤ 24 h, bei 30,8 % (8/26) ≤ 3 Tage und bei 19,2 % (5/26) ≤ 5 Tage angestrebt. Bei 15,4 % (4/26) der Kliniken bleibt die Intubation regelhaft über 5 Tage hinaus bestehen. Die Entscheidung zur Tracheotomie wird bei 96,2 % (25/26) der Zentren stets individuell getroffen, wenn dies aufgrund von Schluckstörung, prolongiertem Weaning oder persistierender Vigilanzminderung als klinisch notwendig eingeschätzt wird. Bei einem Zentrum (3,9 %, 1/26) wird die Indikation zur Tracheotomie nach mindestens einem erfolglosen Extubationsversuch gestellt. Die Methode der Wahl ist in den meisten Zentren mit 77 % (20/26) die Dilatationstracheotomie. Während Tracheotomien bei 19,2 % (5/26) der Kliniken bereits innerhalb der ersten 7 Tage nach Hemikraniektomie durchgeführt werden, erfolgt die Tracheotomie in 80,8 % der Kliniken innerhalb bzw. nach 14 Tagen.

### Bereitschaft zur Teilnahme an klinischen Studien

Zweiundzwanzig von 26 Zentren (84,6 %) zeigten ihre Bereitschaft, an klinischen Studien zum postoperativen neurointensivmedizinischen Management und insbesondere der Dauer der postoperativen Sedierung und Beatmung teilzunehmen.

## Diskussion und Zusammenfassung

### Heterogenität bestehender Behandlungskonzepte

Nach kritischer Indikationsstellung zur Hemikraniektomie steht das postoperative Management im Fokus eines optimierten Behandlungskonzeptes von Patienten/-innen mit einem malignen Mediainfarkt. Die Ergebnisse dieser deutschlandweiten Umfrage spiegeln eine bemerkenswerte Heterogenität in der Behandlungspraxis von Patienten/-innen mit malignem Mediainfarkt und Hemikraniektomie wider. Dies betrifft zum einen grundsätzliche Festlegungen (wie die Anlage einer ICP-Sonde und die regelhafte Durchführung zerebraler Kontrollbildgebungen), das postoperative Management (Sedierungs‑/Intubationsdauer, Anlage Trachealkanüle) als auch die Herangehensweise an das Monitoring des intrakraniellen Druckes (invasiv per ICP-Sonde/klinisch nach Sedierungsrücknahme/nach bildgebendem Entscheid) sowie das unterschiedliche Ausmaß der Anwendung dieser Tools zur Abschätzung der Schwellungsdynamik. Obwohl es in den letzten Jahren teilweise eine generelle Umorientierung beispielsweise bezüglich der Verwendung von Sedativa und Osmotherapeutika gab, scheint weiterhin ein gewisses Maß an Unsicherheit hinsichtlich deren postoperativen Einsatzes zu bestehen. Die wissenschaftliche Evidenz der teils divergenten Herangehensweisen insbesondere zur postoperativen Sedierungsrücknahme mit zügigem Weaning gegenüber einer prolongierten Analgesie, Sedierung und Beatmung ist aktuell unzureichend.

Die Implementierung von Standards in der Intensivmedizin hat sich beispielsweise in den Bereichen Sepsis, Weaning oder Sedierung bezüglich des Therapieziels und klinischer Outcomes als effektiv erwiesen [1, 27, 35]. Standardisierte Konzepte mit Leitlinienadhärenz und Integration eines SOP-basierten Vorgehens in der Neurointensivmedizin wurden in Umfragestudien in den letzten Jahren mehrfach thematisiert. Dabei waren klinikinterne Protokolle je nach Krankheitsbild bei 80–100 % der befragten Neurointensivstationen im Einsatz, wobei bezüglich Sedierung und Weaning nur bei 30–40 % standardisierte Vorgehensweisen bestanden [3]. Eine generelle Leitlinienadhärenz wurde in einer Umfrage von 2014 zufolge bei 41 % der Intensivstationen mit 75 % geschätzt und Standardverfahren wurden in über 80 % angewandt [16]. Für die Therapie des Status epilepticus führten in einer aktuelle Umfrage 38 % der befragten Intensivstationen keine Standardarbeitsanweisung (SOP), 33 % verfolgten der Auswertung zufolge zumindest vergleichbare Behandlungsansätze [17]. Auch im Hinblick auf die Intensivtherapie von Subarachnoidalblutungen besteht mangels Standardisierung der Wunsch nach Konsens [10].

### Therapeutische Sedierung oder frühe Extubation

Der multimodale Ansatz einer therapeutischen Sedierung mit dem Ziel einer Senkung des intrakraniellen Druckes findet nach dem Ergebnis der Umfrage auch bei dem malignen Mediainfarkt nach erfolgter Hemikraniektomie breite Anwendung. Zu inhalativen Sedativa und Thiopental, welche zu einem geringen Prozentsatz eingesetzt werden, liegen bisher beim malignen Mediainfarkt keine generellen Empfehlungen vor [2]. Bedingt durch eine anhaltende Druckerhöhung im parenchymatösen Gewebe kommt es zu einer Minderperfusion mit Dysfunktion der zerebrovaskulären Autoregulation. Konsekutiv resultieren sekundäre Ischämien mit kritischer Sauerstoffunterversorgung und letztlich Zunahme des zytotoxischen und später vasogenen Ödems [6]. Theoretisch soll die zur Therapie des intrakraniellen Druckes eingesetzte therapeutische Sedierung den Stoffwechselgrundumsatz für Sauerstoff und konsekutiv das zerebrale Blutvolumen reduzieren und dadurch den intrakraniellen Druck senken (Monro-Kellie-Doktrin; [2]). Demgegenüber steht die Befürchtung, einen hinreichenden zerebralen Perfusionsdruck (CPP) nicht aufrechterhalten zu können [12]. Bei einem schweren Schädel-Hirn-Trauma, welches ebenfalls mit der Ausbildung eines raumfordernden Hirnödems einhergeht, konnte dabei keine Überlegenheit eines bestimmten Sedativums aufgezeigt werden [28].

Insgesamt fehlt ein einheitliches und konkretes postoperatives Konzept für das Sedierungsmanagement einschließlich der Planung von Aufwachversuchen zur klinischen Beurteilung [9, 15, 31]. Dies spiegelt auch das Ergebnis der Umfrage wider. Die Rücknahme der Sedierung wird in den überwiegenden Fällen individuell entschieden, beispielsweise orientierend an dem individuellen Weaningkonzept oder anhand des klinischen Verlaufes. Auffällig sind die standardmäßig sehr späten Extubationszeitpunkte mit stellenweise > 5 Tagen nach dem Ereignis bei etwa 15 % der teilnehmenden Kliniken. Im Falle einer prolongierten Beatmungsentwöhnung mit ggf. schlechter Tubustoleranz besteht das Potenzial, eine bedarfsangepasste Analgesie und Sedierung mit dem primären Ziel der Stressreduktion anzuwenden und bei ohnehin zuvor erfolgter therapeutischer Sedierung eine kumulative zentralnervöse Depression zu riskieren [6, 11]. Dabei steigt mit zunehmender Sedierungsdauer das Risiko sekundärer Komplikationen, bspw. einer ventilatorassoziierten Pneumonie oder eines schweren Delirs [11]. Mit der Hemikraniektomie ist bereits die entscheidende therapeutische Maßnahme erfolgt, sodass eine unmittelbare postoperative Beendigung der Analgesie und Sedierung sowie frühe postoperative Extubation begründbar wäre, wie es von einigen der teilnehmenden Zentren durchgeführt wird. Anhand welchen Vorgehens ein günstigerer klinischer Verlauf erzielt werden kann, ist unter Berücksichtigung der aktuellen Studienlage nicht klar.

### ICP-Sonde, Osmotherapeutika und therapeutische Kühlung

Die Anlage einer ICP-Sonde ist eine individuelle Entscheidung. Gemessen an der Invasivität der Operationsprozedur erscheint die Anlage einer ICP-Sonde trotz parenchymatöser Bougierung ein relativ gering-invasiver Eingriff. Zwar spiegelt die Messung des intrakraniellen Druckes per ICP-Sonde nur lokale Gegebenheiten im Gewebe wider, diese kann jedoch wichtige zusätzliche Informationen beispielsweise zur Schwellungsdynamik und zum Erfolg einer hyperosmolaren Therapie des intrakraniellen Druckes oder passageren Hyperventilation bzw. Lagerungsmaßnahmen liefern und in Kenntnis des mittleren arteriellen Druckes dem Monitoring des CPP dienen [29, 30]. Gemäß gültiger Leitlinienempfehlung wird bei Patienten/-innen mit erhöhtem intrakraniellem Druck die intravenöse Applikation kreislaufdepressiver Wirkstoffe stets unter Kontrolle des mittleren arteriellen Druckes (und ggf. ICP-Kontrolle) empfohlen [2]. Weiterhin wird darauf hingewiesen, dass eine Messung des intrakraniellen Druckes nach Entlastungstrepanation sinnvoll sein kann [12]. Allerdings kommt eine ICP-Sonde nach vorliegender Umfrage nur in knapp 25 % der Kliniken standardisiert bei Hemikraniektomie zur Anwendung.

Der überwiegende Anteil der an der Umfrage teilnehmenden Kliniken verwendet standardisiert Osmotherapeutika nach einem festen Schema. Gemäß aktueller Empfehlungen kann bei pathologischer Erhöhung des intrakraniellen Druckes die Gabe osmotisch wirksamer Substanzen erwogen werden, wobei eine prophylaktische Gabe nicht empfohlen wird [12]. Es konnte gezeigt werden, dass eine Osmotherapie den intrakraniellen Druck positiv beeinflussen kann [22]. Allerdings fehlen multizentrische randomisierte kontrollierte Studien, die den klinischen Nutzen einer hyperosmolaren Therapie bei einem raumfordernden Mediainfarkt belegen. Eine rezente retrospektive Analyse von 219 Patienten/-innen mit malignem Mediainfarkt, die eine hyperosmolare Therapie mit Mannitol erhielten, weist darauf hin, dass eine akute schwere Nierenschädigung eine häufige und potenziell irreversible Komplikation der hyperosmolaren Therapie sein kann [20]. Auch kann die diuretische Wirkung zu einem intravasalen Volumenmangel mit konsekutiver Hypotonie und Abfall des CPP führen [12]. Daher sollte der Einsatz von Osmotherapeutika stets im Sinne einer Risiko-Nutzen-Abwägung kritisch geprüft werden und ein entsprechendes Monitoring gewährleistet sein.

Der seltene (3,9 %, 1/26) Einsatz einer therapeutischen Kühlung nach Hemikraniektomie mit dem Ziel der Normothermie wirft Fragen im Hinblick auf das Temperaturmanagement auf Neurointensivstationen auf. Insbesondere bleibt offen, inwieweit apparative Temperaturmanagementsysteme (z. B. Oberflächenkühlung mittels Arctic Sun^TM^ oder intravaskulär mittels Thermoguard XP®) den befragten Neurointensivstationen zur Verfügung stehen.

### Früh- oder Spättracheotomie

Bei Patienten/-innen mit schwerem Schlaganfall stellen dekompressive Hemikraniektomie und ein erfolgloser Extubationsversuch die wichtigsten Prädiktoren für die Notwendigkeit einer Tracheotomie dar [19]. Dabei korreliert das Risiko einer erfolglosen Extubation mit der Schwere der Dysphagie [32]. Sind die Extubationskriterien aus verschiedenen Gründen (z. B. anhaltende Vigilanzminderung, verminderte Schutzreflexe) im postoperativen Setting nicht erfüllt, stellt sich die Frage der Weiterführung der täglichen Prüfung vs. der unmittelbaren Tracheotomie. Dieser Diskurs könnte die heterogenen Angaben zu den angestrebten Extubationszeitpunkten erklären.

In einer kürzlich veröffentlichen randomisierten kontrollierten Studie zum Timing der Tracheotomie (Stroke-related Early Tracheostomy vs. Prolonged Orotracheal Intubation in Neurocritical care Trial 2, SETPOINT2) hatte die frühe Tracheotomie (≤ 5 Tage nach Intubation) im Vergleich zur späten Tracheotomie (≥ 10 Tage nach Intubation) keinen signifikanten Effekt auf das funktionelle Outcome nach 6 Monaten [4]. Dennoch wird auch in der vorliegenden Umfrage mehrfach das Kriterium des zügigen Weanings als Begründung für eine Sedierungsrücknahme benannt. Tatsächlich zeigte sich in SETPOINT2 eine Tendenz zu geringerem Bedarf an Sedativa unter den Patienten/-innen, die zu einem frühen Zeitpunkt tracheotomiert wurden. Dies widerspricht dem Ansatz einer therapeutischen Sedierung im Kontext einer Therapie des intrakraniellen Druckes, welche erst nach hinreichendem Schwellungsrückgang beendet wird. Das Ausbleiben potenziell protektiver Effekte einer nur kurzzeitigen therapeutischen Sedierung könnte den Benefit einer frühen Tracheotomie überlagern. Inwieweit die Durchführung einer Tracheotomie, wie im Ergebnis der Umfrage, auch bis zu 14 Tage warten darf, bleibt offen, scheint aber den Ergebnissen der SETPOINT2-Studie zufolge auch im Zeitintervall ≥ 10 Tage nicht mit relevanten Nachteilen einherzugehen. Damit steht das Zeitoptimum der Trachealkanülenanlage in Konkurrenz zum Sedierungskonzept weiterhin zur Diskussion.

### Versorgungslandschaft deutscher (Neuro‑)Intensivstationen

Gemäß dem Verzeichnis der DGNI sind in Deutschland 235 Neurointensivstationen gelistet, was prinzipiell eine gute Ausgangslage für die Behandlung neurointensivpflichtiger Patienten/-innen bietet [34]. Zu erwähnen ist allerdings, dass diese Auflistung in hohem Maße auch interdisziplinäre Intensivstationen mit ausschließlich fachneurologischer oder -neurochirurgischer Konsiliarpräsenz enthält. Dies korrespondiert mit einer Analyse aus dem Jahr 2017, welche aufgrund der nur wenigen eigenständigen Neurointensivstationen in Deutschland (39 der 320 berücksichtigten Intensivstationen) erheblichen Nachbesserungsbedarf für eine hinreichende und fachgerechte Patientenversorgung anzeigte [5]. Dies ist insofern von Relevanz, als dass gerade die Behandlung von Patienten/-innen mit malignem Mediainfarkt einer hohen neurologischen als auch neurochirurgischen Expertise bedarf.

Nach unserer Analyse der G‑BA-Qualitätszahlen lässt sich feststellen, dass die dekompressive Hemikraniektomie am häufigsten in schlaganfallversorgenden Ballungsräumen durchgeführt wird. Da diese großteils auch die IGNITE-Zentren umfassen, kann unsere Umfrage in Bezug auf versorgungsstarke Regionen mit erwartbar optimierten Abläufen prinzipiell als repräsentativ gelten. Zudem waren überwiegend Universitätskliniken mit eigenständigen Neurointensivstationen bzw. mit guter Anbindung an die Neurochirurgie und Neuroradiologie an der Befragung beteiligt. Diese Selektion der beteiligten Kliniken kann dabei das Überwiegen bestimmter Entscheidungsabläufe erklären, wie beispielsweise das Fehlen einer oberen Altersgrenze für die Indikationsstellung einer Hemikraniektomie. Vor diesem Hintergrund ist die bestehende Heterogenität der postoperativen Intensivtherapie in besonderem Maße beachtenswert.

Kleinere Zentren waren den Ergebnissen unserer Umfrage zufolge nicht beteiligt. Basierend auf den G‑BA-Qualitätszahlen lassen sich aber auch Schwankungen von Hemikraniektomien zugunsten niedrigvolumiger Schlaganfallzentren erkennen. Dies könnte für eine Umverteilung bzw. selektive Verlegung von Patienten/-innen zur operativen Therapie sprechen. Die Frage, ob an kleineren Zentren und auch interdisziplinären Intensivstationen mit ausschließlicher neurologischer Konsiliarpräsenz eine noch größere Heterogenität besteht, kann anhand der Umfrage nicht beantwortet werden. Damit bleibt auch offen, ob die weiterführende Behandlung nach Hemikraniektomie an einem überregionalen neurologischen Zentrum erfolgen soll oder alternativ auch telemedizinisch vermittelt werden kann.

### Limitationen

Die vorliegende Umfrage bildet einen Ausschnitt der neurointensivmedizinischen Versorgungpraxis in unterschiedlichen Zentren ab. Dieser kann zur orientierenden Bestandsaufnahme herangezogen werden, erhebt jedoch nicht den Anspruch, die deutschlandweite Behandlungspraxis im Gesamtquerschnitt abzubilden und kann damit per se nicht als repräsentativ gelten. Um die Frage der externen Validität der Umfrage zu beantworten, bedarf es daher einer deutschlandweiten Studie mit Berücksichtigung aller an der Behandlung von Patienten/-innen nach dekompressiver Hemikraniektomie beteiligten Intensivstationen. Aufgrund der überwiegenden Teilnahme von Universitätskliniken könnte eine Verzerrung zu höheren Versorgungsstufen hin bestehen. Ein Responsebias kann nicht ausgeschlossen werden. Vorstellbar ist eine positive Antworttendenz bedingt durch ein grundlegendes Bestreben der bestmöglichen Versorgung in der eigenen Klinik. Die Umfrage spiegelt folglich die Sichtweise auf die aktuelle Praxis im eigenen Klinikum wider und beinhaltet u. U. subjektive Schätzwerte. Durch die anonymisierte Teilnahme konnte dennoch eine hohe Rücklaufquote (67,4 %) erreicht werden, allerdings gestattet dies keine Rückschlüsse zu regionalen Unterschieden der Behandlungspraxis. Zuletzt besteht das Risiko einer systematischen Verzerrung der Ergebnisse zu den untersuchten Themenbereichen der Umfrage, da teilnehmende Kliniken diese vereinzelt nicht beantwortet hatten.

## Fazit für die Praxis

- Die aktuelle heterogene Versorgungsrealität spiegelt die geringe Evidenz zum postoperativen Management und insbesondere der Dauer der postoperativen Analgesie und Sedierung sowie Beatmung bei Patienten/-innen mit malignem Mediainfarkt nach erfolgter Hemikraniektomie wider.
- Einheitliche Therapie- und Qualitätsstandards sind notwendig.
- Um eine optimale neurointensivmedizinische Versorgung von Patienten/-innen mit malignem Mediainfarkt und Hemikraniektomie zu gewährleisten, muss die Anwendbarkeit standardisierter Konzepte auf die deutschlandweite Versorgung geprüft werden.
- Überregionale Netzwerkstrukturen (ggf. unter Einbeziehung telemedizinischer Konzepte) könnten möglicherweise unausgewogene Versorgungslandschaften kompensieren.

### Supplementary Information




