# Supplementary material for: Postoperative management following decompressive hemicraniectomy for malignant middle cerebral artery infarction—A German nationwide survey study
Source: Nervenarzt. 2023 May 4;94(10):934–43. [Article in German] doi: 10.1007/s00115-023-01486-4 (PMC10157548; doi:10.1007/s00115-023-01486-4)
Supplement: Supplementary file 1 [file 115_2023_1486_MOESM1_ESM.pdf]

Bestandsaufnahme
zur Vorgehensweise nach Hemikraniektomie
bei malignem Mediainfarkt

Fragen zur Organisationstruktur Ihrer Klinik

1. Welche Art Krankenhaus ist ihre Klinik?

Bitte wählen Sie nur eine der folgenden Antworten aus:

- ◯ Universitätsklinik
- ◯ Klinik in kommunaler Trägerschaft
- ◯ Klinik in kirchlicher Trägerschaft
- ◯ Klinik in privater Trägerschaft
- ◯ Sonstiges

2. Verfügt ihr Standort über eine Neurologie mit 24h Verfügbarkeit?

Bitte wählen Sie nur eine der folgenden Antworten aus:

- ◯ Ja
- ◯ Nein

3. Verfügt ihr Standort über eine Neurochirurgie?

Bitte wählen Sie nur eine der folgenden Antworten aus:

- ◯ Ja
- ◯ Nein

4. Verfügt ihr Standort über eine interventionelle Neuroradiologie?

Bitte wählen Sie nur eine der folgenden Antworten aus:

- ◯ Ja
- ◯ Nein

5. Über welche Bettenkapazitäten verfügen sie?

[ ]

6. Wie ist die Struktur ihrer Intensivstation?

Bitte wählen Sie nur eine der folgenden Antworten aus:

- ◯ Eigenständige Neurologische Intensivstation
- ◯ Interdisziplinäre Neurologische/Neurochirurgische Intensivstation
- ◯ Anästhesiologische Intensivstation
- ◯ Sonstiges

[ ]

Fragen zur Versorgungssituation von Patienten mit malignem Mediainfarkt

7. Wie viele Patienten mit malignem Mediainfarkt wurden in ihrer Einrichtung in den letzten 5 Jahren durchschnittlich pro Jahr betreut?

Bitte wählen Sie nur eine der folgenden Antworten aus:

- ◯ ≤ 6 Patienten
- ◯ ≤12 Patienten
- ◯ > 12 Patienten
- ◯ Die Anzahl schwankt deutlich (Angabe pro Jahr)

Wenn „Die Anzahl schwankt deutlich (Angabe pro Jahr)“ bei 7. ► 8. Wie viele Patienten mit malignem Mediainfarkt wurden in ihrer Einrichtung in den letzten 5 Jahren betreut?

[ ]

9. Wie ist die Datengrundlage der genannten Zahlen?

Bitte wählen Sie nur eine der folgenden Antworten aus:

- ◯ Schätzung
- ◯ Controlling

10. Besteht in ihrer Klinik eine feste SOP bezüglich der Versorgung von Patienten mit einem malignen Mediainfarkt?

○ Ja
○ Nein

Wenn „Ja“ bei 10. ► 11. Wenn ja, wären Sie bereit, das entsprechende Dokument zur Verfügung zu stellen?

○ Ja
○ Nein

12. Sie können ihre SOP direkt hier hochladen. Vielen Dank!

Fragen zur Indikationsstellung einer Hemikraniektomie bei Patienten mit malignem Mediainfarkt

13. Legen Sie die Indikationsstellung zur Durchführung einer Hemikraniektomie bei Patienten mit einem malignem Mediainfarkt anhand der DESTINY-Kriterien fest (klinische Symptomatik für einen Infarkt im Stromgebiet der Art. cerebri media, NIHSS score >14 (nicht-dominante Hemisphäre) oder >19 (dominante Hemisphäre), Bewusstsein nach NIHSS 1a > 0, Symptombeginn < 48 Stunden)?

Bitte wählen Sie nur eine der folgenden Antworten aus:

○ Ja
○ Nein

14. Haben Sie in ihrer Klinik für die Indikationsstellung zur Durchführung einer Hemikraniektomie bei Patienten mit einem malignen Mediainfarkt eine obere Altersgrenze festgelegt?

Bitte wählen Sie nur eine der folgenden Antworten aus:

○ Ja
○ Nein

Wenn „Ja“ ► 15. Was ist ihre obere Altersgrenze für die Durchführung einer Hemikraniektomie?

Bitte geben Sie Ihre Antwort hier ein:

16. Besteht an Ihrer Klinik ein festgelegtes Zeitfenster ≤ 48 Stunden nach Symptombeginn für die Durchführung einer Hemikraniektomie bei malignem Mediainfarkt?

Bitte wählen Sie nur eine der folgenden Antworten aus:

◯ Ja
◯ Nein

Wenn „Ja“ bei 16. ► 17. Wird eine Hemikraniektomie bei einem Zeitfenster > 48 Stunden in Ausnahmefällen durchgeführt?

Bitte wählen Sie nur eine der folgenden Antworten aus:

◯ Ja
◯ Nein

Fragen zum chirurgischen Vorgehen

18. Wird intraoperativ standardisiert eine Hirndrucksonde angelegt?

Bitte wählen Sie nur eine der folgenden Antworten aus:

◯ Ja
◯ Nein

19. Wird direkt postoperativ standardisiert eine Kontrollbildgebung durchgeführt?

Bitte wählen Sie nur eine der folgenden Antworten aus:

◯ Ja
◯ Nein

20. Wird am Folgetag nach der Operation standardisiert eine Kontrollbildgebung durchgeführt?

Bitte wählen Sie nur eine der folgenden Antworten aus:

- ◯ Ja
- ◯ Nein

Wenn „Ja" bei 20. ► 21. Zu welchem(n) Zeitpunkt(en) wird standardisiert eine Kontrollbildgebung durchgeführt?

Bitte geben Sie Ihre Antwort hier ein:

Fragen zur Therapie nach erfolgter Hemikraniektomie

22. Haben Sie an Ihrer Klinik ein standardisiertes Vorgehen für die Dauer der Analgosedierung nach erfolgter Hemikraniektomie bei malignem Mediainfarkt?

Bitte wählen Sie nur eine der folgenden Antworten aus:

- ◯ Ja
- ◯ Nein

Wenn „Ja" bei 22. ► 23. Wie lange wird die Analgosedierung in der Regel standardisiert fortgeführt?

Bitte wählen Sie nur eine der folgenden Antworten aus:

- ◯ Beendigung in der Regel direkt nach erfolgter Entlastungsoperation
- ◯ ≤ 24 Stunden
- ◯ ≤ 3 Tage
- ◯ ≤ 5 Tage
- ◯ regelhaft Fortführung der Analgosedierung > 5 Tage

Wenn „Nein" bei 22. ► 24. Nach welchen Kriterien entscheiden Sie sich zur Sedierungsrücknahme?

Bitte geben Sie Ihre Antwort hier ein:

25. Erfolgt regelhaft eine Kontrollbildgebung vor geplanter Beendigung der Analgosedierung?

Bitte wählen Sie nur eine der folgenden Antworten aus:

- ◯ Ja
- ◯ Nein

26. Welche Applikationsform nutzen Sie bevorzugt zur Analgosedierung nach Hemikraniektomie?

Bitte wählen Sie nur eine der folgenden Antworten aus:

- ◯ i.v.-Sedativa
- ◯ inhalative Sedativa

Wenn „i.v. Sedativa" bei 26. ► 27. Welche Wirkstoffe verwenden Sie?

Bitte wählen Sie alle zutreffenden Antworten aus (Mehrfachnennungen möglich):

- ◯ Midazolam
- ◯ Andere Benzodiazepine
- ◯ Etomidat
- ◯ Thiopental
- ◯ Sufentanil
- ◯ Fentanyl
- ◯ Ketamin
- ◯ Propofol
- ◯ Dexmedetomidin
- ◯ Sonstiges:

28. Verwenden Sie standardisiert intravenöse Osmotherapeutika zur medikamentösen Hirndrucktherapie?

Bitte wählen Sie nur eine der folgenden Antworten aus:

- ◯ Ja
- ◯ Nein

Wenn „Ja“ bei 28. ► 29. Gibt es ein festes Schema, nach dem intravenöse Osmotherapeutika verabreicht werden?

Bitte wählen Sie nur eine der folgenden Antworten aus:

- ◯ Ja
- ◯ Nein

Wenn „Ja“ bei 28. ► 30. Welche Wirkstoffe verwenden sie?

Bitte wählen Sie alle zutreffenden Antworten aus (Mehrfachnennungen möglich):

- ◯ Mannitol
- ◯ Hyperosmolares Natrium
- ◯ Sonstiges:

Wenn „Ja“ bei 29. ► 31. Nach welchen Kriterien entscheiden Sie über die Gabe intravenöser Osmotherapeutika?

Bitte wählen Sie nur eine der folgenden Antworten aus:

- ◯ Hirndruckmessung via ICP-Sonde
- ◯ Klinische / bildgebende Befunde (z.B. Anisokorie, progrediente Herniation)
- ◯ Sonstiges

32. Erfolgt in ihrer Klinik standardisiert eine therapeutische Kühlung der hemikraniektomierten Patientin bei malignem Mediainfarkt?

Bitte wählen Sie nur eine der folgenden Antworten aus:

- ◯ Ja
- ◯ Nein

Wenn „Ja“ bei 32. ►33. Welche Zieltemperatur wird angestrebt?

Bitte wählen Sie nur eine der folgenden Antworten aus:

- ◯ Normothermie
- ◯ <37 °C
- ◯ <35 °C

◯ <33 °C

34. Hat ihre Klinik an der DEPTH-SOS-Studie teilgenommen?

Bitte wählen Sie nur eine der folgenden Antworten aus:

◯ Ja
◯ Nein

35. Bezüglich der Intubationsdauer, über welchen Zeitraum verbleiben Patienten nach erfolgter Hemikraniektomie bei malignem Mediainfarkt in der Regel aus klinischer Indikation bei Ihnen intubiert?

Bitte wählen Sie nur eine der folgenden Antworten aus:

◯ Extubation wird in der Regel direkt nach erfolgter Entlastungsoperation angestrebt
◯ ≤ 24h
◯ ≤ 3 Tage
◯ ≤ 5 Tage
◯ regelhaft Intubation > 5 Tage

36. In welcher Situation und wann versorgen Sie Patienten, die eine Hemikraniektomie aufgrund eines malignen Mediainfarktes erhielten, mit einer Trachealkanüle?

Bitte wählen Sie nur eine der folgenden Antworten aus:

◯ regelhaft Tracheotomie nach Hemikraniektomie bei malignem Mediainfarkt
◯ Entscheidung zur Tracheotomie immer individuell, wenn dies aufgrund von Schluckstörung, prolongiertem Weaning oder persistierender Vigilanzminderung als klinisch notwendig eingeschätzt wird
◯ in der Regel erst nach mindestens einem erfolglosen Extubationsversuch

37. Welche Tracheotomie-Methode wird bei ihnen nach erfolgter Hemikraniektomie bevorzugt angewandt?

Bitte wählen Sie nur eine der folgenden Antworten aus:

◯ konventionell chirurgisch
◯ Dilatationstracheotomie

38. Zu welchem Zeitpunkt wird in der Regel eine Tracheotomie durchgeführt?

Bitte wählen Sie nur eine der folgenden Antworten aus:

- ◯ < 7 Tage
- ◯ ≤ 14 Tage
- ◯ > 14 Tage

Fragen zur Bereitschaft zur Teilnahme an einer randomisierten Studie

39. Wären Sie zur Teilnahme an einer randomisierten Studie bereit, die bei Patienten mit einem malignen Mediainfarkt den klinischen Nutzen einer frühen (≤24h) vs. späteren (>24h) Beendigung der Analgosedierung nach erfolgter Hemikraniektomie untersucht?

Bitte wählen Sie nur eine der folgenden Antworten aus:

- ◯ Ja
- ◯ Ja, wenn folgende Bedingungen erfüllt sind ...
- ◯ Nein
- ◯ Nein, weil…

Bitte schreiben Sie einen Kommentar zu Ihrer Auswahl

Tabelle 1: Antwortverhalten der Kliniken zu den Themenbereichen der Umfrage

	Themenbereiche*					
	1	2	3	4	5	6
Anzahl Fragen	6	6	5	4	17	1
Kliniken (n)	29	28	27	27	26	26

*Fragen 1. zur Organisationstruktur der Klinik, 2. zur Versorgungssituation von Patienten mit malignem Mediainfarkt, 3. zur Indikationsstellung einer Hemikraniektomie bei Patienten mit malignem Mediainfarkt, 4. zum chirurgischen Vorgehen, 5. zur Therapie nach erfolgter Hemikraniektomie, 6. zur Bereitschaft zur Teilnahme an einer randomisierten Studie
